# Comparison of the effects of core stability and whole-body electromyostimulation exercises on lumbar lordosis angle and dynamic balance of sedentary people with hyperlordosis: a randomized controlled trial

**DOI:** 10.1186/s13102-024-00879-5

**Published:** 2024-04-23

**Authors:** Mohammad Hamzeh Shalamzari, Mohammad Amin Henteh, Alireza Shamsoddini, Ali Ghanjal

**Affiliations:** 1 Department of Sports Injury and Biomechanics, Faculty of Sport Sciences and Health, University of Tehran, Tehran, Iran; 2 Exercise Physiology Research Center, Life Style Institute, Baqiyatallah University of Medical Sciences, Tehran, Iran; 3 Health Management Research Center, Baqiyatallah University of Medical Sciences, Tehran, Iran

**Keywords:** Exercise training, Lordosis, Dynamic balance, Electrical stimulation therapy, Spine

## Abstract

**Background:**

Hyperlordosis is an excessive inward curvature of the lumbar spine that affects spinal function. The aim of this study was to compare the effects of core stability exercises (CSE), Whole-Body Electromyostimulation (WB-EMS), and CSE Plus on the Lumbar lordosis angle and dynamic balance in sedentary people with hyperlordosis.

**Methods:**

In a parallel randomized controlled trial study, seventy five untrained male adults with hyperlordosis, recruited from clinics of sports medicine and corrective exercise centers in Tehran, were randomly assigned to four groups: CSE (*n* = 19), WB-EMS (*n* = 18), CSE Plus (*n* = 18), and control Group (CG) (*n* = 20). The CSE group performed Core stability exercises, the WB-EMS group followed a Whole-body electromyostimulation combined training protocol, and the CSE Plus group engaged in a combined program protocol (CSE with the WB-EMS vest), and the control group only participated in activities of daily living. Anthropometric parameters and outcomes, including the lordosis angle and dynamic balance, were assessed before and after a six-week training program. A flexible ruler was used to measure the angle of lordosis, and the Y balance test was employed to evaluate the dynamic balance.

**Results:**

The results indicated that the lordosis angle improved in both the CSE and CSE Plus groups compared to the CG in the post-test (*P* = 0.017, *P =* 0.024). However, there were no significant differences observed between the other group pairs. Additionally, a significant difference in dynamic balance was found between the CSE Plus group and the CG in the post-test (*P* = 0.001), while no significant differences were observed between the other group pairs. Furthermore, within-group test results demonstrated that lumbar lordosis angle and dynamic balance variables significantly improved in the post-test compared to the pre-test stage (*P* < 0.05).

**Conclusions:**

The two CSE and CSE Plus training protocols are effective as training methods for correcting certain parameters and physical deformities, including lumbar lordosis. Furthermore, the CSE Plus group demonstrated a positive impact on improving dynamic balance. Consequently, it is highly recommended that individuals with hyperlordosis can benefit from the exercises of the present study, especially CSE Plus exercises along with other rehabilitation exercises.

**Trial registration:**

The trial was registered at Thai Clinical Trials Registry (TCTR20221004011, registration date: 04/10/2022).

## Background

The spine is a vital component of the human body, with a distinctive structure that varies among individuals. It can be influenced by both heredity and environmental conditions, and may undergo changes over time due to various factors such as inactivity. Spinal deformities affect the body’s skeletal framework and are more common than other deformities. Taweetanalarp and Purepong (2015) stated that the lumbar lordosis angle is significantly higher in sedentary individual and those with a higher body mass index (BMI) [1]. One of the most important parts of the spine is the lumbar curve, and an excessive anterior curvature in the lumbar arch is termed lumbar hyperlordosis.

Lumbar hyperlordosis is caused by weakness and tightness in the muscles and soft tissues of the pelvis, abdomen, and spine, called core areas [2]. According to the kinesiopathology model, changes in the function of the movement system in one area of the body can lead to the development of musculoskeletal disorders over time [3]. Therefore, following the occurrence of muscle imbalance in the lumbopelvic region (dominance of force couple caused by lumbar extensor and hip flexors due to prone to tightness over force couple caused by hip extensor and abdominal muscles due to prone to elongation and weakness), in people with lumbar hyperlordosis, the anterior pelvic tilt occurs, lumbar curve increases, and core stability is impaired [2, 4]. In this regard, Garcia et al. (2006) stated that insufficient function of the abdominal muscles can increase the angle of the spinal curvature due to chain reactions [5].

On the other hand, due to the close relationship between the lumbar vertebrae and the pelvis through the sacrum, any change in pelvic position changes the amount of lumbar arch, and consequently, any increase or decrease in the angle of the arch affects the body balance [4]. Evidence suggests that muscles function in the core region has an important role in the lumbopelvic balance and spinal deformities [5]. Weakness in the core stabilizer muscles following hyperlordosis and its association with pelvic changes can be a potential factor in reducing balance.

The use of early corrective approaches may prevent some complications and further long-lasting deformity of the spine in sedentary people with hyperlordosis and correct the deformity. Manipulation, postural retraining, use of brace or orthosis, and exercise therapy are the most important therapeutic-corrective methods [6, 7]. Among the mentioned methods, corrective exercise is one of the most common corrective methods which its effects on tissue changes, muscle activity, and the severity of deformities has already been investigated in the literature.

Core stability exercises, have created a approach in the field of corrective exercise and rehabilitation [8]. Barr et al. (2005) proposed core stability exercises increase lumbar stability, coordination between extensor and flexor muscles’ contraction, and neuromuscular control function [9]. Willardson(2007) stated that there is a significant relationship between core stability exercises and proper alignment of the spine [10].

Today, a variety of more recent methods are being explored to determine greater effectiveness. As mentioned, stability exercises have an effective role in improving neuromuscular adaptations and can change the muscle recruitment pattern and important role in retraining of muscles’ activity. In addition to core stability exercises, exercises with Electrical Muscle Stimulation (EMS) devices and special vests have been recently employed to stimulate different muscle groups simultaneously through generating electrical pulses [11]. This training method is called whole-body electromyostimulation (WB-EMS). In this method, weak muscles can be stimulated and strengthened without moving the joints, which makes it an excellent choice in cases of injury, atrophy or muscular dystrophy [11]. In fact, WB-EMS‌ enhances the muscles activation through superficial electrical stimulation as well as nerve impulses emitted from the brain. It is claimed that EMS vests create an electric field and as a result contractions of muscle fibers through the electrode and pulses sent to the skin [12]. Filipovic et al.(2016) showed that the use of WB-EMS can be effective in increasing the muscular strength of elite soccer players [13]. Therefore, WB-EMS can be used as a new tendency to complement conventional training and be an alternative training option for the less active community who are not able to perform physical activity for any reasons.

Given the relatively high prevalence and the negative consequences that may result from postural deformities, as well as the limited experimental evidence in this area, the question arises that if core stability exercises and WB-EMS can correct lumbar hyperlordosis? Since the core muscles have a great impact on maintaining and controlling balance, further researches should be done on whether these exercises may improve the balance of people with lumbar hyperlordosis, which is itself a factor in reducing balance.

It is hypothesized that CSE, WB-EMS, and the CSE Plus would reduce the lumbar hyperlordosis angle and improve dynamic balance. Therefore the aim of this study was to compare of the effects of core stability and whole-body electromyostimulation exercises on lumbar lordosis angle and dynamic balance of sedentary people with hyperlordosis.

## Methods

The CONSORT statement (updated guidelines for reporting parallel group randomized trials) [14] was used as a guideline to report this randomized controlled trial.

### Study design

This study was a multicenter, parallel randomized controlled four-arm trial with an allocation ratio of 1:1:1:1, conducted in Tehran. The procedures followed were in accordance with Human Research Ethics committee of the Baqiyatallah University of Medical Sciences (IR.BMSU.REC.1399.489), and the Helsinki Declaration of 1975, as revised in 2000. The study’s Clinical Trails Registry number is TCTR20221004011.

### Participants

**Fig. 1:**
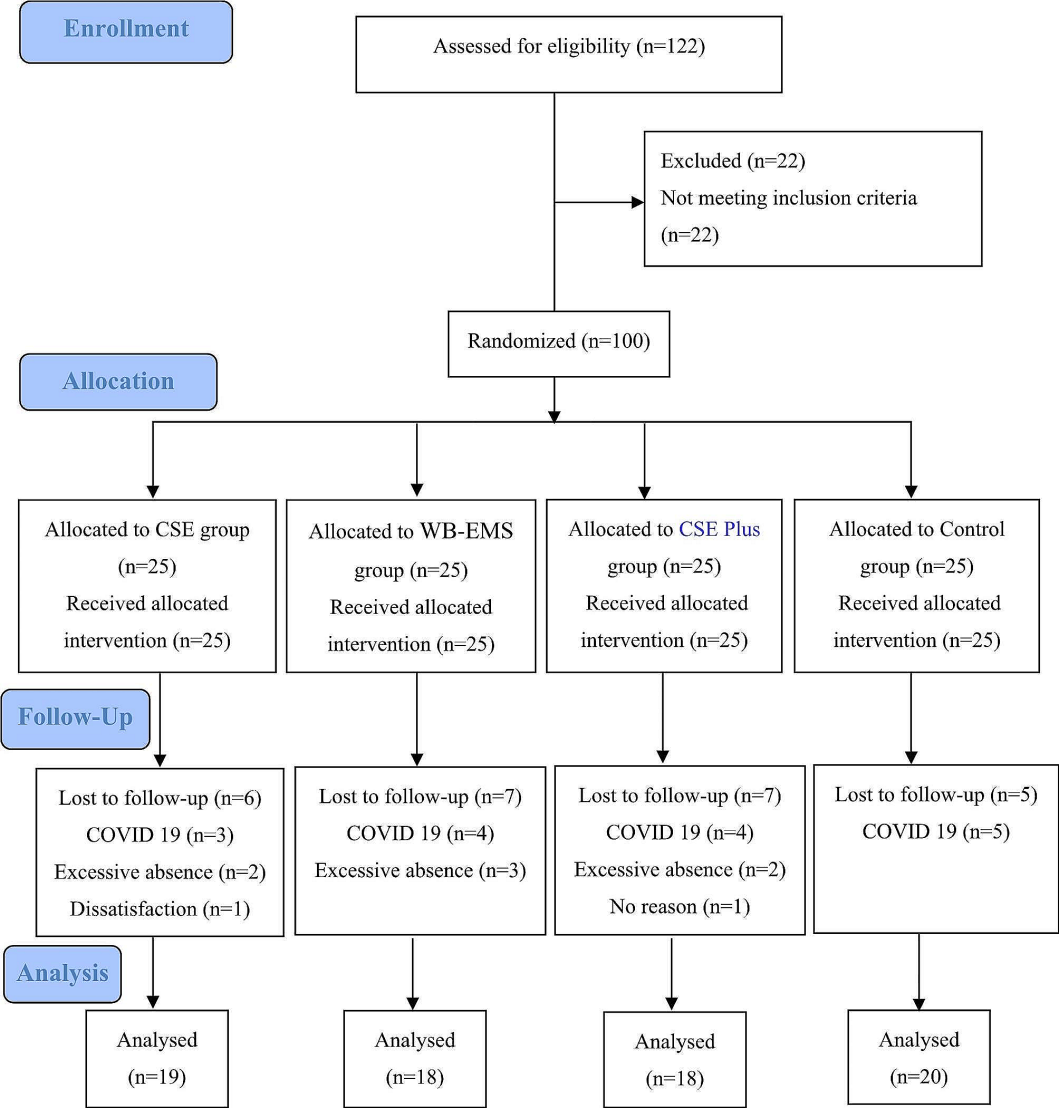
Consort flow diagram

Seventy-five sedentary people with lumbar hyperlordosis were recruited between December 2020 and March 2021 from clinics of sports medicine and corrective exercise centers in Tehran, Iran, through printed and social-media advertisements. The data were collected at the Laboratory of Sport Injures at the university of Tehran and Baqiyatallah University of Medical Sciences. The inclusion criteria were: 1- Age between 20 and 40 years, 2- Not having a regular and moderate to high intensity exercise in the last six months based on IPAQ-SF questionnaire [15], 3- No history of fracture, surgery or joint disease in the spine and 4- Having more than 40 degrees lumbar lordosis [16]. The exclusion criteria were: 1- incomplete implementation of the training program (three alternate sessions or two consecutive sessions), 2- absence on pre-test or post-test, 3- having pain and inability to perform exercises, and 4- the observation of related pathological symptoms, such as heart diseases, cancer, tumors, previous surgery, etc. Then, the participants gave their informed consent and completed the baseline assessments. In order to assess and perform the exercises, the subjects were asked to be present at a designated time and place while observing health guidelines on COVID-19 (Fig. 1).

### Randomization and blinding

An independent researcher, who was not involved in the treatment protocol or assessments, used the random number function of Excel to generate randomization sequences. The random numbers were placed in opaque envelopes, which were subsequently opened in sequence by study staff, who knew the sample. Participants were randomly assigned to training and control groups: (1) control, (2) core stability exercises (CSE), (3) whole-body electromyostimulation (WB-EMS), and (4) CSE Plus. Participants and researchers were unblinded to group assignment and were aware of the Exercise interventions components, but the study’s hypotheses were not disclosed to the participants. The participants were assessed by the same blinded assessor at baseline (pre-test) and 6-week (post-test) and the assessor and statistician were blinded to participant allocation.

### Interventions

Participants in the intervention groups attended two sessions per week for 6 weeks while the control group did not receive any training intervention between the test sessions. All participants were informed about the process of the study and signed written consent before the study commenced. They voluntarily participated in this study and were allowed to discontinue co-operation whenever desired. None of the subjects had prior experience or knowledge concerning CSE or EMS training.

#### Core stability exercises protocol

These exercises considering the exercise science principles (such as overload, volume, intensity and integrity), are based on the core stability exercise program proposed by Jeffrey and include five levels (Table 1) [17]. Exercises start from level one, which includes static contractions in a fixed position, and level 2 exercises include holding static contractions in a stable environment. Level 3 exercises include holding static contractions in an unstable environment (Swiss ball), and. Level 4 exercises include the dynamic movements in an unstable environment, and finally level 5 exercises include dynamic movements with resistance in an unstable environment [17]. The volume of exercise and increasing the load of each exercise were determined based on a correct execution of exercises and pressure exerted on each subject in the previous session.

#### Whole-body electromyostimulation combined training protocol

**Fig. 2:**
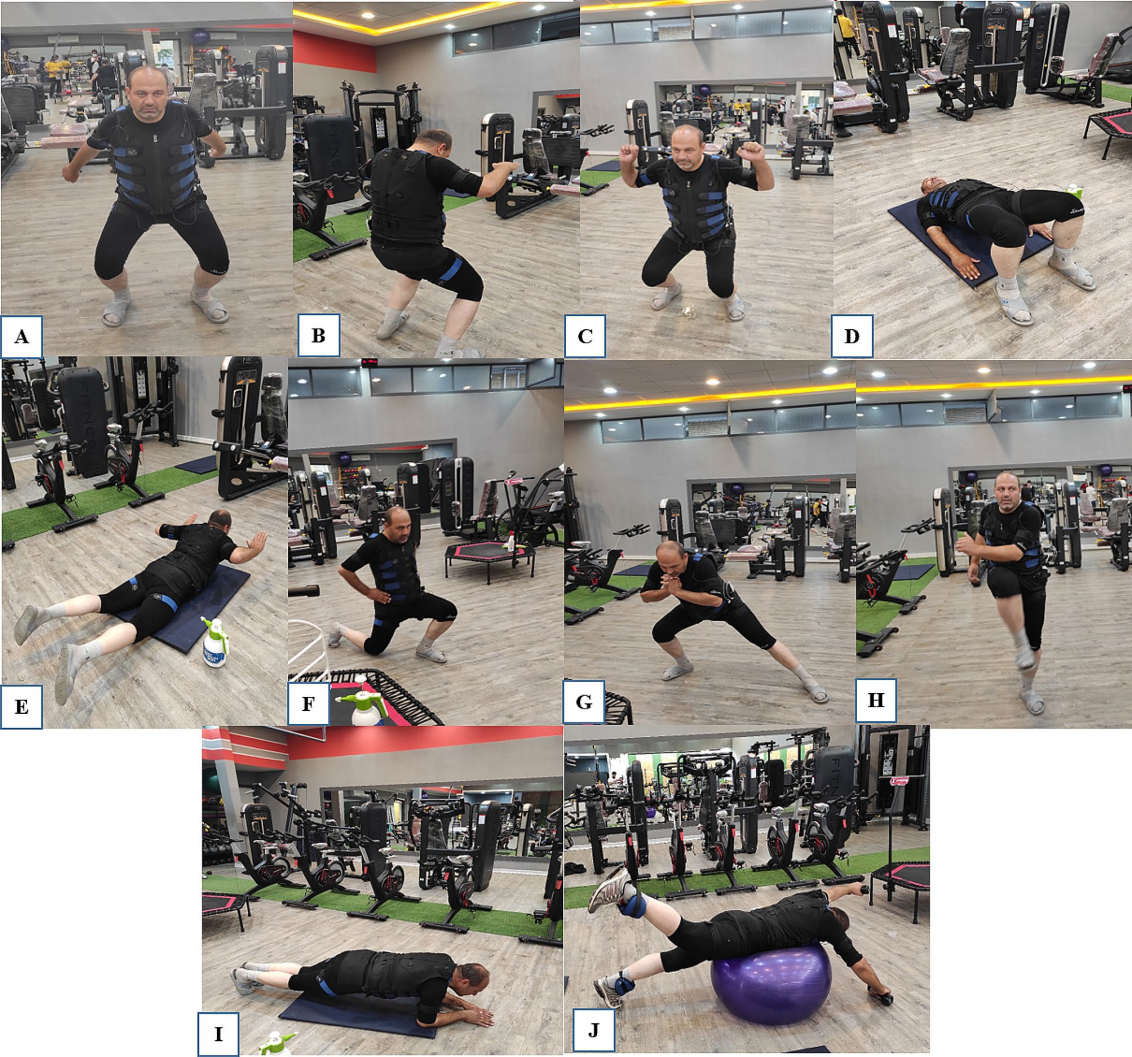
WB-EMS training: **A** Dynamic squat. **B** Dynamic trunk flexion with retracted arms. **C** Shoulder press combined with the squat. **D** Bridge. **E** Superman back. **F** Static forward lunge. **G** Dynamic side lunge. **H** Diagonal crunches in standing position. **I** Front plank. **J** Dynamic trunk extension with Swiss-ball

WB-EMS training was conducted using the Miha Bodytec 2 WB-EMS device (Miha Bodytec, Augsburg, Germany), which is wirelessly controlled by a tablet under the guidance of an instructor. It stimulates different muscle groups simultaneously by generating electrical pulses. The WB-EMS protocol used and Stimulation applied in this study obtained from recent studies [11, 18, 19].The participants performed two 20-minute exercise sessions per week for six weeks (Fig. 2). The WB-EMS protocol was performed according to Table 2.

#### CSE plus combined program protocol

These exercises included the CSE protocol (Table 1), which performed with the WB-EMS vest. An electric current of 85 Hz was alternately applied using the 6-second activation and the 4-second rest and a pulse width of 350 μm (Table 2).

**Table 1:** Core stability exercises program

Type / Exercises	
Training A	abdominal drawing-in and pull the lower back to the floor(Static contraction)
Training B	Supine bridges
Training C	Crunches
Training D	Plank with opposite arm and leg lift
Training E	Cat-Cow Stretch whit contraction abdominal muscles
Training F	Lowering and lifting legs in supine position
Training G	Reverse Crunch
Training H	Crisscross Crunch
Training I	single leg glute bridge
Training J	Single leg Bridge On Swiss Ball
Training K	Swiss ball crunch
Training L	Rotational Knee Tuck on an Swiss Ball

*Warm up and stretching and, jogging, running, or cycling (10–15 min)

**Number of Repeats: 12–20 s in static exercises or 20–45 Repetitions in dynamic exercises, Number of Sets: 3, Rest Period: 1–2 min.

***cool down: 10 min.

**Table 2:** Whole-body electromyostimulation program

Type / Exercises	
Training A	Dynamic squat
Training B	Dynamic trunk flexion with retracted arms
Training C	Shoulder press combined with the squat
Training D	Bridge
Training E	Superman back
Training F	Static forward lunge, left and right
Training G	Dynamic side lunge, left and right
Training H	Diagonal crunches in standing position, left and right
Training I	Front plank
Training J	Dynamic trunk extension with Swiss-ball

* Parameters: Stimulation frequency: 85 Hz; Impulse duration: 6 s; Impulse break: 4 s; Pulse breadth: 350 µs; Impulse type: Bipolar; Duration: 20 min.

**Warm up and stretching and, jogging, running, or cycling (10–15 min)

***Number of Repeats: 10–15, Number of Sets: 3, Rest Period: 1 min.

****Cool down: 10 min.

### Outcome assessment

First, the demographic data were collected from the participants. The dependent variables, the primary (lumbar lordosis angle) and secondary (dynamic balance) outcome measures were then assessed employing the below mentioned procedures.

#### Primary outcome measures

The primary outcome of the study was the lumbar lordosis angle which was assessed using a 0.4 m flexible ruler. Several studies have documented strong validity and reliability of this tool [20, 21]. To measure lumbar lordosis, two bony landmarks (spinous process of the T12 as the beginning of the arc and the S2 as the end of the arc) were marked with sticky removable red points while the participants stood on their feet and looked toward the opposite wall. The flexible ruler was then placed on the lumbar area, between the two landmarks S2 and T12, to form a lumbar lordosis. Without changing the shape of the ruler, its pattern was drawn on a white paper, and the T12 and S2 points were marked. To calculate the lumbar lordosis angle, the T12 and S2 points were measured by a straight line (L), and another line (H) was drawn perpendicular to the center of the arc. The lordosis angle was calculated by placing the values of L and H lines in the following formula [20, 22]:

$$ \theta = 4\left[ {Arctan\left( {\frac{{2H}}{L}} \right)} \right]$$

#### Secondary outcome measures

**Fig. 3:**
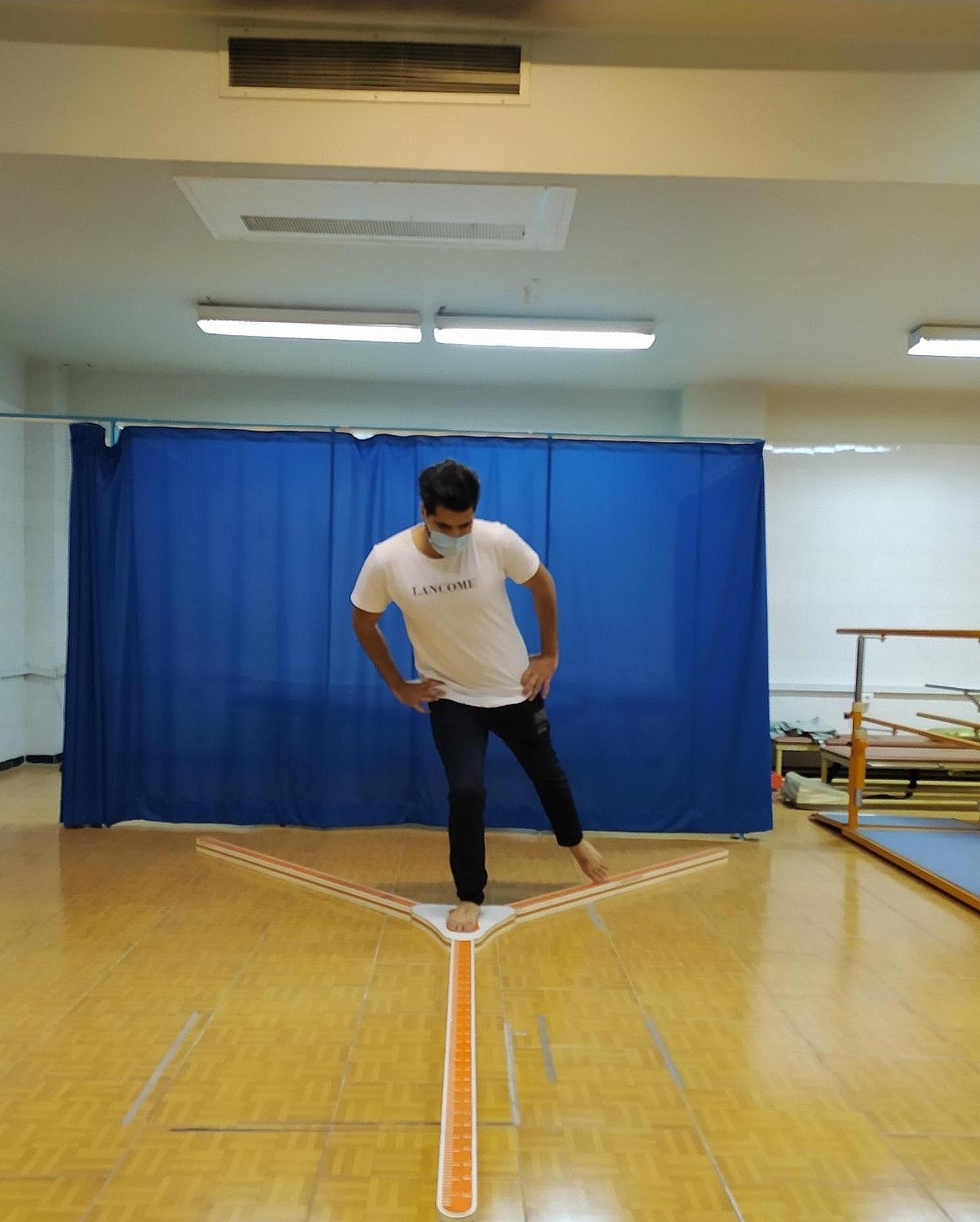
Y balance test

The secondary outcomes included the dynamic balance assessment. Y dynamic balance test was used to measure dynamic balance in the present study [23]. The researchers reported excellent Interrater reliability (ICC = 0.97–0.99) and moderate to excellent Intrarater (test-retest) reliability (ICC = 0.68–0.90) [24]. Also the results of the previous research indicated that the Y balance test is a valid measure (values 0.05–0.72) in the assessment of dynamic balance [23]. As shown in Fig. 3, the subjects stood on a stationary platform in the center of the Y balance kit with their dominant foot. The subject then attempted to move the indicators plate of all three anterior, posteromedial and Posterolateral directions with their other foot as far away as possible. To record total score, the subjects performed three trials in each direction, and a rater measured the mean of these three trials. To normalize the calculated values for each direction, the mean was divided by the limb length (in centimeters) and then multiplied by 100. The sum of three values calculated in each direction was subsequently divided by three [23, 24].

### Sample size

The sample size was calculated using G*Power 3.1. For a repeated (2 × 4) ANOVA (within and between interactions, f = 0.22, a = 0.05) a minimum group size of 96 persons was calculated (power 0.958). Assuming an approximate dropout rate of 25%, a final total sample size of 122 was calculated. Finally, out of 122 test subjects, only 100 met the inclusion criteria, and 25 subjects did not complete the 6 weeks training and were removed from the study. In the end, the data from 75 subjects was included in the evaluation (Fig. 1).

### Statistical analysis

The data related to the subjects are analyzed in two sections of descriptive and inferential statistics in SPSS version 22. Group differences between the test times were calculated using repeated (2 × 4) ANOVA. Comparisons of pairs were calculated post hoc based on Bonferroni. Differences between the groups (CG, CSE, WB-EMS, and CSE Plus) were calculated for pre- and post-tests using Univariate ANOVA with post hoc based on Tukey. The significance level was set at 5% (α = 0.05).

## Results

The results of descriptive statistics for individual’s demographic characteristics are presented in Table 3. The distribution of all outcome measures in the four groups was determined to be normal based on the values of the Shapiro-Wilk test. No significant differences were observed between the four groups with respect to demographic data (*P* > 0.05).

**Table 3:** Participants characteristics at baseline testing.*†

Group	Number	Height (m)	Weight (kg)	Age (y)	Body mass index(kg. m^2^)
**CSE**	19	1.79 ± 0.06	84.47 ± 4.43	29.16 ± 5.43	26.38 ± 1.81
**WB-EMS**	18	1.79 ± 0.05	87.00 ± 5.20	29.50 ± 5.33	27.15 ± 2.16
CSE Plus	18	1.78 ± 0.05	86.06 ± 5.59	27.78 ± 4.68	27.13 ± 2.08
CG	20	1.77 ± 0.06	85.50 ± 6.15	29.40 ± 5.89	27.11 ± 2.03

*CSE group = core stability exercises; WB-EMS group = whole-body electromyostimulation; CSE Plus group = CSE with the WB-EMS vest; CG = control group.

†Values are presented as mean ± SD.

**Table 4:** The results of Repeated Measures Analysis of Variance (ANOVA)

Variable	Source	Total Sum of Squares	df	Mean Square	F	Sig.	Effect Size
Lordosis	**Time**	116.82	1	116.82	185.32	0.0001^**^	0.723
	Time * Group	56.80	3	18.93	30.03	0.0001^**^	0.559
	**Group**	90.52	3	30.17	1.58	0.201	0.063
Dynamic Balance	**Time**	647.17	1	647.17	935.01	0.0001^**^	0.929
	Time * Group	292.86	3	97.62	141.03	0.0001^**^	0.856
	**Group**	306.12	3	102.04	1.54	0.210	0.061

^†^Values are presented as mean ± SD. p values were obtained by Analyze of Variance (2 times × 4 groups)

^**^P ≤ 0.05 significant difference within and between groups in post intervention.

According to Table 4, the combined analysis of variance (ANOVA) showed a significant interaction between the group and the research stages (time × group) in the values of the lordosis angle variable [df = 3, F = 30.03, *P* = 0.0001].

Furthermore, the results of ANOVA showed a significant interaction between the group and the research stages (time × group) in the values of dynamic balance variable [df = 3, F = 141.03, *P* = 0.0001] (Table 4).

According to the results obtained from combined ANOVA and the significance of the interaction of time × group to lordosis angle and dynamic balance (*P* < 0.05), the data was analyzed again with post hoc.

The result of Tukey’s post hoc analysis in lordosis angle variable showed a significant difference between the groups CSE vs. CG (*P* = 0.017) and CSE Plus vs. CG (*P* = 0.024), but no difference between the other group pairs (CSE vs. WB-EMS: *P* = 1.000; CSE vs. CSE Plus: *P* = 1.000; WB-EMS vs. CSE Plus: *P* = 1.000; WB-EMS vs. CG: *P* = 0.487).

The result of Tukey’s post hoc analysis in dynamic balance variable showed a significant difference between only the groups CSE Plus vs. CG (*P* = 0.001), but no difference between the other group pairs (CSE vs. WB-EMS: *P* = 1.000; CSE vs. CSE Plus: *P* = 0.074; CSE vs. CG: *P* = 0.733; WB-EMS vs. CSE Plus: *P* = 0.295; WB-EMS vs. CG: *P* = 0.242).

The Bonferroni’s post-hoc test was used for intra-group comparisons. The results of Bonferroni’s post-hoc test are presented in Table 5.

**Table 5:** Banferoni test results related to within-group differences

Variable	Group	Mean Difference (95% CI) ^¥^	F	Sig.	Effect Size
Lordosis	**CSE**	1.89 ± 0.25 (1.38, 2.40)	54.10	0.0001^**^	0.432
	**WB-EMS**	1.83 ± 0.26 (1.30, 2.36)	47.98	0.0001^**^	0.403
	**CSE Plus**	3.38 ± 0.26 (2.86, 3.91)	163.96	0.0001^**^	0.698
	**CG**	0.05 ± 0.25 (− 0.45, 0.55)	0.04	0.834	0.001
Dynamic Balance	**CSE**	4.77 ± 0.27 (4.23, 5.31)	312.54	0.0001^**^	0.815
	**WB-EMS**	4.29 ± 0.27 (3.74, 4.84)	240.00	0.0001^**^	0.772
	**CSE Plus**	7.64 ± 0.27 (7.09, 8.20)	760.58	0.0001^**^	0.915
	**CG**	0.08 ± 0.26 (− 0.44, 0.60)	0.10	0.752	0.0001

*CSE group = core stability exercises; WB-EMS group = whole-body electromyostimulation; CSE Plus group = CSE with the WB-EMS vest; CG = control group.

^**¥**^ Values are presented as mean ± SD.

**P ≤ 0.05 significant difference within groups from baseline to post intervention.

According to Table 5, After 6 weeks of training interventions, a significant improvement was observed in lordosis angle in all three groups of CSE, WB-EMS, and CSE Plus in the post-test compared to the pre-test, but no significant change was observed in lordosis angle in the CG from the pre-test to post-test. Also, a significant improvement was observed in dynamic balance in all three groups of CSE, WB-EMS, and CSE Plus in the post-test compared to the pre-test, but no significant change was observed in the CG in any of the factors related to dynamic balance from the pre-test to post-test.

## Discussion

The purpose of this study was to determine the effect of CSE, WB-EMS and CSE Plus on lumbar lordosis angle and dynamic balance in male with hyperlordosis. The results of the present study showed a significant reduction in lordosis angle after 6 weeks of CSE and CSE Plus interventions in male with hyperlordosis. In addition, the dynamic balance of the subjects significantly increased only in the CSE Plus group. Although the dynamic balance in the CSE and WB-EMS groups improved after 6 weeks, these values were not statistically significant.

In the CSE and CSE Plus exercise groups, a significant improvement was observed in lordosis angle compared to the CG, which had a similar effect compared to the corrective exercises used in previous studies. Babakhani reported a positive effect of core muscle exercises with physioball balls on balance and lordosis curve of trainable mentally retarded female students [25]. They concluded that 12 weeks of core stability training using a physioball ball could be an effective way to reduce lumbar lordosis.

Ludwig et al. conducted a study evaluating the effect of WB-EMS on body posture and trunk muscle strength in untrained individuals, which is consistent with the results of the present study. They concluded that 10 weeks of WB-EMS did not significantly alter the parameters of physical condition, including lumbar lordosis [11].

The main cause of musculoskeletal disorders such as hyperlordosis is muscle imbalance [26].Kendall suggested that the abdominal muscles become weak and elongated in hyperlordosis, which need strengthening [27]. In the present study, no muscle examination was performed; however, it is worth noting that the muscles of the core area, such as the transverse abdominis and the internal oblique muscles, are connected to the thoracolumbar fascia. Strengthening these muscles may be effective in correcting the deformity through correcting the length-stress relationship. The goal of core stability training is to improve muscle strength or endurance, along with enhancing neuromuscular coordination and muscle fiber recruitment [28]. Since hyperlordosis deformities involve muscle imbalance and disturbances in stress-strain relationships [29], CSE may help improve lordosis curve angle through strengthening weak muscles, improving neuromuscular coordination and also modifying the stress-strain relationship [28, 30]. 

Typically, electrodes integrated in the vest can stimulate the following muscles: the chest muscles (particularly the pectoralis major muscle) trapezius muscles (especially middle trapezius), the latissimus dorsi muscle, and abdominal muscles (especially rectus abdominis), humerus, thighs and gluteal muscles [11].

Ludwig et al. analyzed adolescents with poor physical condition and concluded that isolated exercises of the muscles affecting the pelvis (Rectus Abdominis, Gluteus Maximus, Biceps Femoris, and Semitendinosus) improve pelvic position [31]. Since lumbar lordosis is directly related to anterior pelvic tilt, the angle of lordosis decreased possibly following the reduction of anterior pelvic tilt due to the strengthening of target muscles, including the abdominal muscles and thigh extensors.

Ludwig stated that unspecific WB-EMS training activates muscle groups in the trunk, pelvis, and legs, i.e. simultaneous stimulation by large surface electrodes activates all the underlying muscle fibers [11]. Therefore, it is possible that WB-EMS and CSE exercises, in addition to strengthening the core global muscles, also strengthen the lumbar local and deep muscles, such as multifidus muscles and transversus abdominis, which leads to increased coordination and development of core stability and reduction of extra force on the spine, thus reduction in the depth of lumbar lordosis. It seems that CSE Plus exercises, including the simultaneous combination of WB-EMS exercises and CSE, increase the recruitment of motor units and increase the frequency and coordination of neural impulse caused by electrical stimulation, which can improve motor learning and neuromuscular coordination and thus create balance between tight and weak muscles [32].

In fact, CSE Plus exercises act as a coordinator of the agonist and antagonist muscles. Body posture in general is based on the muscle interaction of agonist and antagonist. An imbalanced relationship between these causes the body to shift from a neutral position to a weak posture [11]. Ludwig et al. found that the WB-EMS trains agonist and antagonist muscles to a similar extent and generates positive effects on strength parameters of the back, influencing LBP and the potential imbalance of the muscle groups [11]. Therefore, it is possible that WB-EMS training can also reduce the lordosis angle by creating muscle balance between agonist and antagonist muscles.

Some studies have shown that spinal deformities can increase the amplitude of changes in balance disorders [33]. In fact, physiological mechanisms in the balance systems of these people change, and erroneous information is sent about the spatial orientation of the body to the brainstem. This information sends inappropriate movement instructions and consequently causing disorder in the body posture. Therefore, reduction in muscle efficiency in sending information, reduction in core muscles coordination, reduction in the proprioception role of these muscles, repetitive injuries in sedentary people, and the presence of deformities cause balance disorder.

The intra-group results indicate the positive effect of three exercises of CSE, WB-EMS, and CSE Plus on the balance index in post-exercise measurements, while showing no change in the CG. But, the inter-group results showed that there was a difference only between the CSE Plus exercises and the control group in the post-test. This difference may be due to changes in proprioception conditions during CSE Plus exercises, which creates more demands on the balance control system than separate CSE or WB-EMS exercises [34]. Consistent with the results of the present study, Trampas et al. positively expressed the immediate effects of combining core stability exercises and clinical massage on dynamic balance performance of patients with chronic specific low back pain [35].

Regarding how combined exercises can effect on balance and postural control, CSE and WB-EMS program possibly improve the efficiency of the neuromuscular system, resulting in the optimal movement of the lumbopelvic complex during the functional motor chain, proper muscle balance, strengthening of proximal stability, and functional strength [36]. These effects lead to optimal performance and increase in the strength of the lower limb muscles, which can better stabilize the muscles and thus neutralize torques produced during the movements, thus the person can maintain his/her balance longer [37].

On the other hand, vibrations produced in WB-EMS exercises also stimulate the large muscles of the body, including the abdominal muscles, erector spinae muscles, the thigh muscles, and the gluteal muscles. Since the vibrations produced increase the muscle activity of almost the whole body, they stimulate the nerve roots and strengthen the neuromuscular system; as a result, they improve the performance of proprioception receptors in areas that affect balance strategies, such as the sacral lumbar region [38].

The dynamic nature of combined CSE with WB-EMS exercises can also improve body posture, balance, coordination, body awareness, flexibility, and strength. Additionally, WB-EMS activates the postural muscles, which often leads to the stimulation of proprioception and the somatosensory system [12]. The CSE also activates some areas of the brainstem, vestibular, and cerebellum system [39], both of which ultimately lead to body and posture control and increased balance.

Performing CSE alone enhances muscle strength and endurance along with improving the relationship between neural control and the musculoskeletal system [40]. However, WB-EMS mechanical vibrations applied to the muscle belly or tendons can especially activate sensory receptors, particularly muscle spindles responsible for detecting changes in muscle length, as well as large muscles [41]. Therefore, in the combination of these exercises, core stability exercises through active contraction and WB-EMS by passive contraction can engage greater muscle activation. Furthermore, these combined exercises can have better impact on the muscle recruitment, proprioception, and neuromuscular system than CSE alone.

### Limitations

The study has some limitations. Firstly, the present study only investigate the age range of 20 to 40 years due to the potential muscle damage caused by WB-EMS overload that may affect adolescents or the elderly. It is also noteworthy that female samples were not available and were not compared with male samples. Secondly, since performing CSE along with WB-EMS improve muscle strength, and muscle strength can affect our variable research, thus not assessing muscle strength is another limitation. Furthermore, our test persons were all inexperienced in WB-EMS training and not actively involved in sports. Even though it is considered one of the strengths of our study, it cannot transfer the results directly to athletes. Since dynamic balance is closely related to proprioception, it is suggested to further investigate the effect of these three types of exercises on proprioception.

## Conclusions

The findings revealed a significant improvement in the lordosis angle and dynamic balance of sedentary individuals with lordosis disorder in the CSE Plus group (CSE with the WB-EMS vest). Thus, performing core stability exercises along with the WB-EMS vest can yield better results than core stability exercises alone for correcting certain parameters and physical deformities, including lumbar lordosis and dynamic balance.

## Data Availability

The datasets used and/or analyzed during the current study are available from the corresponding author on reasonable request.

## Abbreviations

WB-EMS: Whole-Body Electromyostimulation
CSE: Core Stability Exercises
CG: Control Group
BMI: Body Mass Index
EMS: Electrical Muscle Stimulation
IPAQ-SF: The International Physical Activity Questionnaire - Short Form
CSE Plus: Core Stability Exercises with the WB-EMS vest
